# Karyomegalic interstitial nephritis as a rare cause of kidney graft dysfunction: case report and review of literature

**DOI:** 10.1186/s12882-023-03185-3

**Published:** 2023-05-19

**Authors:** Fatma El-Husseiny Moustafa, Eman Nagy, Salwa Mahmoud Elwasif, Mohamed Sobh

**Affiliations:** 1 Pathology department, Faculty of Medicine, Mansoura University, 35516 Mansoura, Egypt; 2 Mansoura Nephrology and Dialysis Unit, Internal Medicine Department, Faculty of Medicine, Mansoura University, El-Gomhuria Street, 35516 Mansoura, Egypt; 3 Urology and Nephrology Center, Faculty of Medicine, Mansoura University, 35516 Mansoura, Egypt

**Keywords:** Karyomegalic interstitial nephritis, Kidney transplantation, Interstitial nephritis

## Abstract

Karyomegalic interstitial nephritis (KIN) is a rare cause of chronic interstitial nephritis characterized by enlarged renal tubular epithelial nuclei. The first case of KIN reported in a kidney graft was in 2019. Here, we report the first case of KIN in 2 brothers receiving kidneys from 2 different unrelated living donors. A male kidney transplant recipient with focal segmental glomerulosclerosis as the original kidney disease presented with graft impairment and proteinuria, and graft biopsy revealed KIN. This patient had a brother who was also a kidney transplant recipient and had one episode of graft impairment and was diagnosed with KIN as well.

## Introduction

Karyomegalic interstitial nephritis (KIN) is a rare cause of chronic interstitial nephritis characterized by enlarged renal tubular epithelial nuclei, and its incidence is less than 1% of renal biopsies [1]. Histologically, renal biopsies of KIN, like other chronic interstitial nephritis, show non-specific interstitial fibrosis, tubular atrophy, varying degrees of glomerulosclerosis, and vascular lesions. The disease hallmark is the presence of large, dysplastic, hyperchromatic nuclei in the epithelial cells lining the proximal and distal renal tubular cells. No immunofluorescence or electron microscopic findings are peculiar to the diagnosis of KIN [2]. The karyomegalic-like cells might also be present in other organs like the liver, brain, lung, and gastrointestinal tract [3]. The first case reported with large dysplastic nuclei in renal tubular epithelium was in 1974; she was a young lady with hepatocellular carcinoma [4]. In 1979, the term KIN was introduced by Mihatsch et al. [5], who described 3 patients with chronic interstitial nephritis and karyomegaly. Less than 100 cases of KIN in native kidneys were mentioned in the literature. However, KIN was first reported in a kidney transplant recipient in 2019 in a patient who received a renal graft from a related donor (his sister) [6]. To the best of our knowledge, this is the first reported case of KIN in which two brothers received kidneys from two different unrelated living donors.

## Case report

**Fig. 1:**
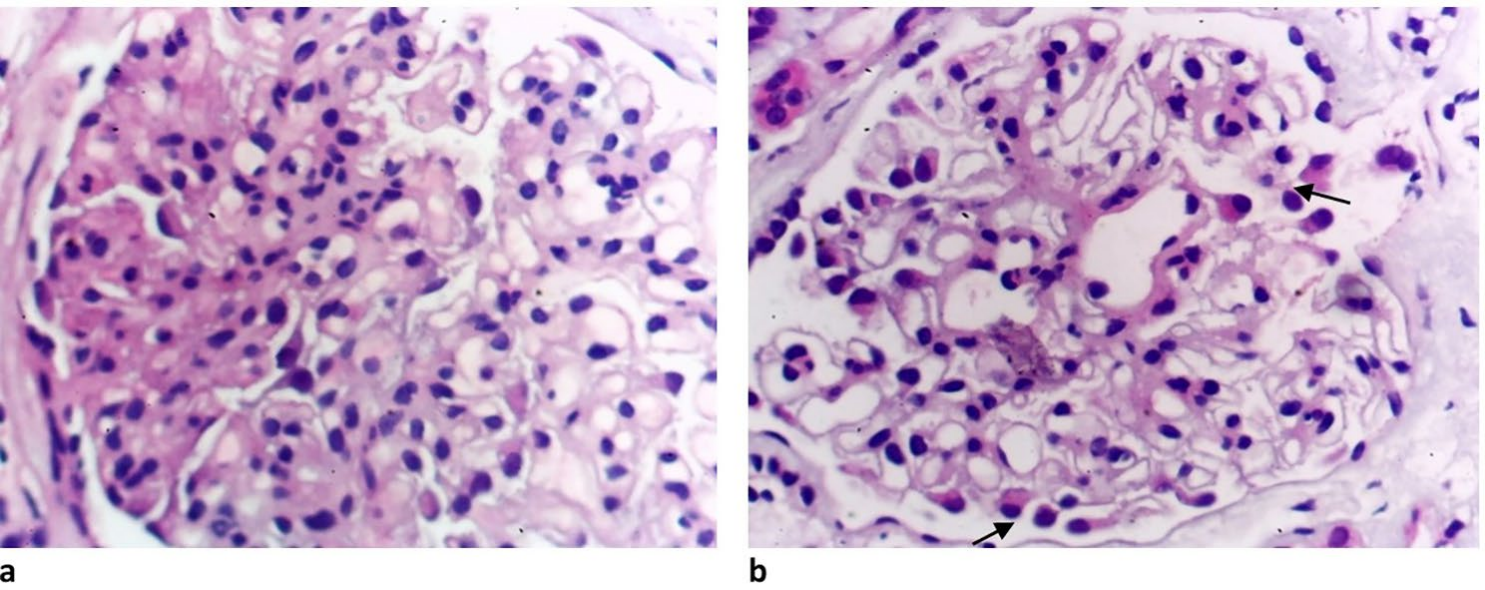
**a**: Focal segmental glomerulosclerosis. (Hx&EX 400). **b**: The other glomeruli show only detached podocytes

**Fig. 2:**
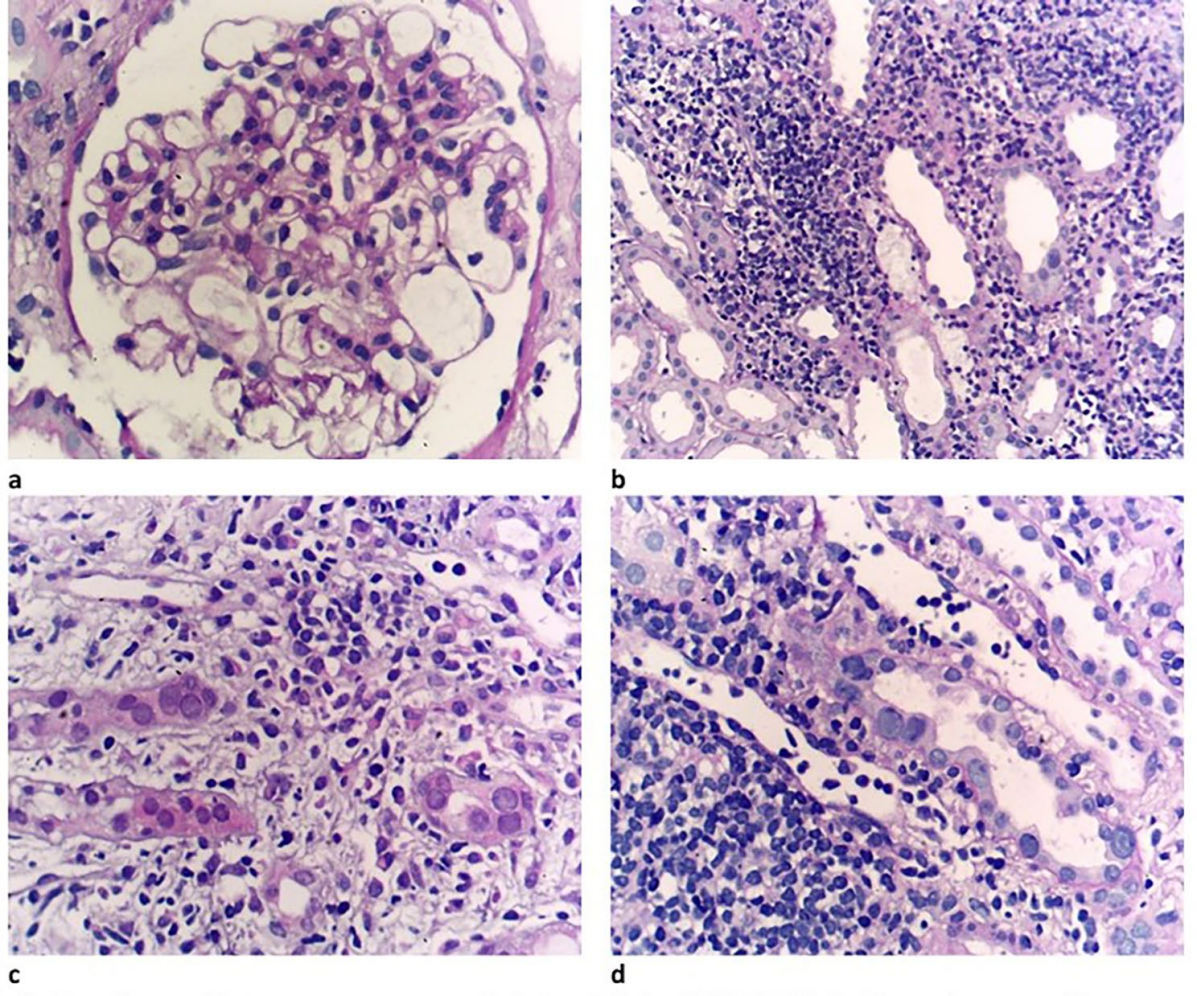
**a**: Glomeruli show aneurysmally dilated capillaries (HX&Ex400). **b**: Dense lymphocytic infiltrate (HX&Ex100). **c**&**d**: Tubular cells have large nuclei. No tubulitis (t0) inspite of the marked dense interstitial infiltrate (i3)

**Fig. 3:**
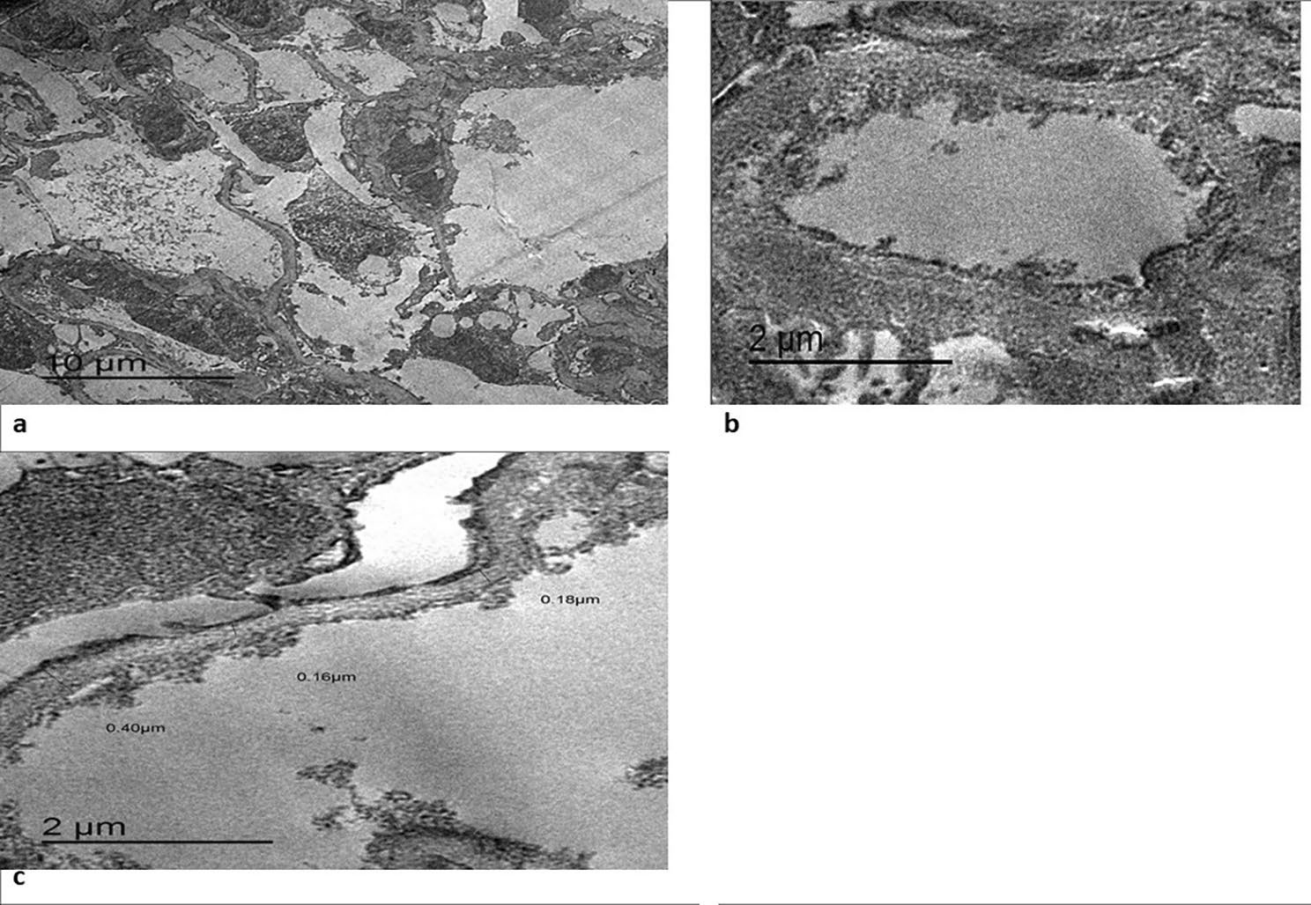
**a**: Detached podocytes. **b**: Lamellated basement membranes. **c**: Thinning & thickening of basement membranes

**Fig. 4:**
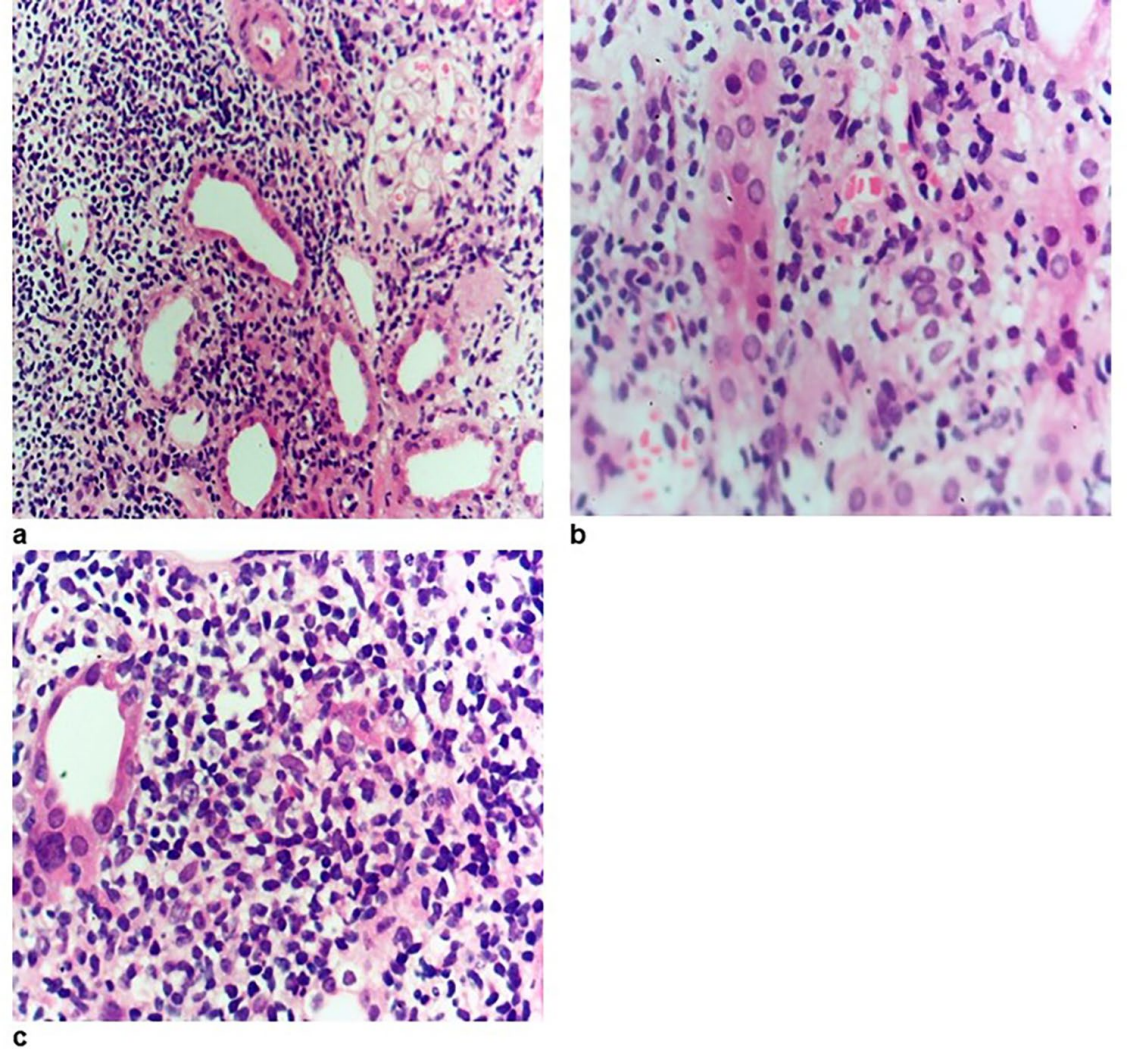
**a**: Dense lymphocytic infiltrate (i3), acute tubular injury. (HX&Ex200). **b**&**c**: Tubular cells have large nuclei (HX&Ex200)

In December 2021, A 37-year-old male Egyptian kidney transplant recipient was referred to our kidney transplant follow-up outpatient clinic. During his follow-up, he was presented with graft impairment (an increase in serum creatinine from 0.6 to 1.2 mg/dl). He was diagnosed with focal segmental glomerulosclerosis (FSGS) (Fig. 1) in 2006, when he was evaluated as a potential kidney donor for his younger brother. He had proteinuria and hypercholesterolemia when he underwent a kidney biopsy, which revealed FSGS, and then he received immunosuppressive therapy in the form of prednisolone and cyclosporine. After that, serum creatinine started to rise until reaching end-stage kidney disease (ESKD) and starting maintenance hemodialysis (HD) in 2014. In October 2021, he received a kidney graft from a 29-year-old unrelated male. He was on maintenance immunosuppressive therapies, which are prednisolone, tacrolimus, and mycophenolic acid, and he had a stable graft function of 0.6 mg/dl. The subsequent two months passed uneventfully, and he presented to us with graft impairment. He was compliant with his maintenance treatment and reported no exposure to nephrotoxic medications, heavy metals, environmental, or agricultural toxins. There was no evidence of any prerenal elements that can cause acute kidney injury. He had a family history of kidney disease: his aunt and maternal cousin were maintaining on HD before their deaths; his father had subnephrotic range proteinuria; and his younger brother had nephrotic syndrome, reaching ESKD, and received a kidney transplant in 2006. The physical examination of the patient was unremarkable. Regarding the laboratory investigations, serum creatinine was 1.2 mg/dl, complete blood count, lipid profile, serum calcium, potassium, and albumin were within normal, arterial blood gases revealed mild metabolic acidosis (PH = 7.30 – bicarbonate 15.7 mmol/L), urine analysis showed pus cells 70–80/HPF, RBCs 15–20/HPF, and albumin +++, urinary albumin creatinine ratio (ACR) was 5900 mg/gm creatinine, urine culture revealed no organism growth, liver enzymes were mildly elevated, and trough level of tacrolimus was within the normal range (9 ng/ml). Graft ultrasound showed normal size and good color perfusion of the graft with a resistive index of 0.7. As the patient presented with graft impairment, leukocyturia, and nephrotic range proteinuria, we are in doubt if the patient had rejection or recurrence. Thus, methylprednisolone 0.5 gm/day for 3 days was started, and a graft biopsy was performed. Graft biopsy showed (Fig. 2a-d) diffuse KIN, acute tubular necrosis, and no evidence of rejection. By electron microscopy, there was podocytopathy with detached podocytes and evidence of collagen IV abnormality (irregular thinning and lamellated basement membranes). (Fig. 3) The investigations for BK nephropathy and cytomegalovirus (CMV) were negative. The patient showed a marvelous response to corticosteroid therapy, serum creatinine dropped to 0.7 mg/dl after 3 days of treatment, and ACR subsided to 600 mg/gm creatinine 3 months after therapy. Returning to the younger brother, a detailed history was taken from him and revealed that he had also experienced an episode of graft impairment 2 months after transplantation. He received at that time pulse methylprednisolone, and a graft biopsy (Fig. 4) showed karyomegalic interstitial nephritis with no evidence of rejection. Urinary BK PCR was negative, and CMV serology was also negative. He also showed a remarkable response to corticosteroid, and serum creatinine decreased to its basal level before this event. Now, after 16 years of transplantation and diagnosis of KIN, the younger brother was stable and had a stable basal creatinine level of 0.9 mg/dl.

## Discussion

Karyomegalic interstitial nephritis, a type of chronic interstitial nephritis, is described when there are enlarged renal tubular epithelial nuclei, interstitial fibrosis, and tubular atrophy. The exact cause of KIN is unknown; however, viral infections, toxic agents, and genetic causes are proposed. Toxic causes include exposure to alkylating agents, heavy metals, and mycotoxins, especially ochratoxin A [7–10]. KIN has been thought to be hereditary, as about half of cases with KIN had a family history of nephropathy [8]. A familial clustering of disease and increased frequency of human leukocyte antigen (HLA)-A9 and HLA-B35 with genetic defects on chromosome 6 were reported [2, 11]. In addition, KIN has been linked to mutations of the Fanconi anemia associated nuclease 1 (FAN1) gene, which is responsible for DNA repair processes [11–13]. Unlike other FAN gene mutations, the FAN1 gene mutation is not associated with Fanconi anemia, risk of malignancy, or developmental anomalies, as the gene is predominant in renal, hepatic, and neuronal tissues [12]. Nevertheless, recent case presentations of a brother and sister with FAN1 mutations and progressive renal failure who developed malignancy after renal transplantation suggest an increased risk of malignancy among patients with kidney transplant recipients and KIN [14]. Recent studies have clarified that KIN is linked to polyploidization of tubular cells, which represents an important mechanism of response to acute kidney injury [15–17]. Airik et al. used a mouse model with knocked-out deoxyribonucleic acid (DNA) repair protein FAN1 and showed that FAN1 inactivation resulted in replication stress and persistent DNA damage. Chronic DNA damage causes cells to fail to complete mitosis and undergo polyploidization, which is characteristic of KIN [16]. Considering KIN is a potential hereditary disease, these genetic abnormalities might increase the patient’s susceptibility to viral and toxic factors that contribute to the disease.

Karyomegalic interstitial nephritis is a slowly progressive chronic interstitial nephritis. Clinically, patients present with mild to moderate renal impairment, reaching ESKD in the fourth or fifth decade of life; however, children may be diagnosed with KIN [7, 18]. Patients with KIN exhibit proteinuria, usually less than 1 gram per day with or without other urinary sediments. About 3/4 of patients display glucosuria, while 2/3 of patients show hematuria. Extrarenal manifestations may include a past history of recurrent respiratory tract infections and abnormalities in liver function tests [2, 3]. Two cases were reported with systemic karyomegaly, where they were diagnosed with KIN and lung manifestations in the form of progressive restrictive lung disease. In these patients, the karyomegalic cells were found in both kidney and lung tissues [19, 20].

Regarding management, there is no specific treatment for slowing the progression of kidney disease in patients with KIN. Corticosteroids were used in trials but without improving renal outcomes [10]. However, one case of a 15-year-old boy diagnosed with KIN after treatment with ifosfamide and cisplatin showed retardation of kidney disease progression after treatment with a moderate dose of corticosteroids [7]. Additionally, in a patient with IgA nephropathy and KIN, treatment with methylprednisolone resulted in stabilization of kidney functions [21].

The original kidney disease in our reported cases was nephrotic syndrome. According to the available slides and data, the eldest brother had FSGS as an original kidney disease, but the details of tubulointerstitium were not available. His graft biopsy showed podocytopathy, which may delineate the early recurrence of FSGS. There are reported cases of coexistence of FSGS and KIN in the literature [18, 22]. These findings bring up the question of whether there is any link between the genetic anomalies of both FSGS and KIN. The FAN1 gene, accused of causing KIN, is found on chromosome 15, while FSGS-induced genes are located on chromosome 19 [22]. However, both renal tubular epithelial cells and podocytes originate from the same tissue, thence genetic association between both diseases cannot be ruled out.

Proteinuria in KIN, as mentioned earlier, is usually less than 1 g/day, but here in the eldest brother, the proteinuria was in the nephrotic range. This can be explained by the presence of glomerular basement membrane (GBM) abnormalities detected by an electron microscope with a diagnosis of collagen IV abnormality. Collagen IV abnormality was linked to Alport syndrome, thin basement membrane disease, or genetically mediated FSGS [23]. The presence of this GBM abnormality may be donor-derived or due to a recurrence of Alport syndrome. The coexistence of hearing loss and FSGS in the eldest brother increased the probability of a diagnosis of Alport syndrome. Most nephrologists believe that patients with Alport syndrome do not develop recurrence of the disease after renal transplantation due to the genetic basis of the disease. However, it has been implied that GBM type IV collagen originates from podocytes recruited from the recipient’s bone marrow-derived cells [24, 25]. In addition, clinical and experimental studies suggested the possibility of recurrence of the Alport syndrome [26–28]. Nevertheless, the patient presented 2 months after transplantation; it is not known if this time is sufficient for the GBM of the graft to alter, and thus donor derived GBM abnormalities are the most likely cause of these abnormalities.

The presence of karyomegalic cells in the renal graft has many differential diagnoses. They present as KIN, BK nephropathy, or T-cell-mediated rejection. In KIN, the nuclei are large, pleomorphic, hyperchromatic, and diffuse in both proximal and distal tubules across the biopsy without intranuclear inclusion and tubulitis. In BK nephropathy, the nuclei are not pleomorphic and contain intranuclear inclusion bodies with focal distribution across proximal and distal tubules and tubulitis. In addition, viral stains, like SV40, are positive. In cell-mediated rejection, the nuclei are not pleomorphic or hyperchromatic and are present in areas of tubulitis without intranuclear inclusion bodies.

The occurrence of KIN in the renal allograft may be due to recurrence, donor-associated disease, or de novo. One of the limitations of this case report is the absence of genetic studies on two brothers, the unavailability of their native biopsies, and the non-performance of SV40 to exclude the diagnosis of BK nephropathy.

## Data Availability

The datasets used and/or analyzed during the current study available from the corresponding author on reasonable request.

## References

[1] Bennani Guebessi N, Karkouri M (2016). Karyomegalic interstitial nephritis. CEN case reports.

[2] Bhandari S, Kalowski S, Collett P, Cooke BE, Kerr P, Newland R (2002). Karyomegalic nephropathy: an uncommon cause of progressive renal failure. Nephrol Dialysis Transplantation.

[3] Monga G, Banfi G, Salvadore M, Amatruda O, Bozzola C, Mazzucco G. Karyomegalic interstitial nephritis: report of 3 new cases and review of the literature. Clin Nephrol. 2006;65(5). 10.5414/cnp65349 16724656

[4] Burry A (1974). Extreme dysplasla in renal epithelium of a young woman dying from hepatocarcinoma. J Pathol.

[5] Mihatsch M, Gudat F, Zollinger H, Heierli C, Thölen H, Reutter F (1979). Systemic karyomegaly associated with chronic interstitial nephritis. A new disease entity?. Clin Nephrol.

[6] Ravindran A, Cortese C, Larsen CP, Wadei HM, Gandhi MJ, Cosio FG (2019). Karyomegalic interstitial nephritis in a renal allograft. Am J Transplant.

[7] Matsuura T, Wakino S, Yoshifuji A, Nakamura T, Tokuyama H, Hashiguchi A (2014). Improvement in karyomegalic interstitial nephritis three years after ifosfamide and cisplatin therapy by corticosteroid. CEN case reports.

[8] Isnard P, Rabant M, Labaye J, Antignac C, Knebelmann B, Zaidan M. Karyomegalic interstitial nephritis: a case report and review of the literature. Medicine. 2016;95(20). 10.1097/MD.0000000000003349 PMC4902386 27196444

[9] Hassen W, Abid-Essafi S, Achour A, Guezzah N, Zakhama A, Ellouz F (2004). Karyomegaly of tubular kidney cells in human chronic interstitial nephropathy in Tunisia: respective role of Ochratoxin A and possible genetic predisposition. Hum Exp Toxicol.

[10] McCulloch T, Prayle A, Lunn A, Watson AR (2011). Karyomegalic-like nephropathy, Ewing’s sarcoma and ifosfamide therapy. Pediatr Nephrol.

[11] Spoendlin M, Moch H, Brunner F, Brunner W, Burger H-R, Kiss D (1995). Karyomegalic interstitial nephritis: further support for a distinct entity and evidence for a genetic defect. Am J Kidney Dis.

[12] Zhou W, Otto EA, Cluckey A, Airik R, Hurd TW, Chaki M (2012). FAN1 mutations cause karyomegalic interstitial nephritis, linking chronic kidney failure to defective DNA damage repair. Nat Genet.

[13] Rejeb I, Jerbi M, Jilani H, Gaied H, Elaribi Y, Hizem S (2021). New familial cases of karyomegalic interstitial nephritis with mutations in the FAN1 gene. BMC Med Genom.

[14] Murray SL, Connaughton DM, Fennelly NK, Kay EW, Dorman A, Doyle B (2020). Karyomegalic interstitial nephritis: cancer risk following transplantation. Nephron.

[15] De Chiara L, Conte C, Semeraro R, Diaz-Bulnes P, Angelotti ML, Mazzinghi B (2022). Tubular cell polyploidy protects from lethal acute kidney injury but promotes consequent chronic kidney disease. Nat Commun.

[16] Airik M, Phua YL, Huynh AB, McCourt BT, Rush BM, Tan RJ (2022). Persistent DNA damage underlies tubular cell polyploidization and progression to chronic kidney disease in kidneys deficient in the DNA repair protein FAN1. Kidney Int.

[17] De Chiara L, Romagnani P (2022). Polyploid tubular cells and chronic kidney disease. Kidney Int.

[18] Radha S, Tameem A, Rao B (2014). Karyomegalic interstitial nephritis with focal segmental glomerulosclerosis: a rare association. Indian J Nephrol.

[19] Tagliente DJ, Voss JS, Peters SG, Aubry MC, Cornell LD, Maleszewski JJ (2014). Systemic karyomegaly with primary pulmonary presentation. Hum Pathol.

[20] Akyürek LM, Hussein A, Nicholson AG, Mauritz N-J, Mölne J (2020). Pulmonary manifestations of systemic karyomegaly. Respiratory Med Case Rep.

[21] Wang Z, Ni X, Zhu S, Yue S (2020). IgA Nephropathy Concomitant with Karyomegalic interstitial nephritis. Am J Med Sci.

[22] Chand MT, Zaka A, Qu H. Association of karyomegalic interstitial nephritis with focal segmental glomerulosclerosis. Autopsy and Case Reports. 2021;11. 10.4322/acr.2021.343 PMC8597774 34805010

[23] Gast C, Pengelly RJ, Lyon M, Bunyan DJ, Seaby EG, Graham N (2016). Collagen (COL4A) mutations are the most frequent mutations underlying adult focal segmental glomerulosclerosis. Nephrol Dialysis Transplantation.

[24] Abrahamson DR, Hudson BG, Stroganova L, Borza D-B, John PLS (2009). Cellular origins of type IV collagen networks in developing glomeruli. J Am Soc Nephrol.

[25] Becker J, Hoerning A, Schmid K, Hoyer P (2007). Immigrating progenitor cells contribute to human podocyte turnover. Kidney Int.

[26] Katsuma A, Nakada Y, Yamamoto I, Horita S, Furusawa M, Unagami K (2018). Long-term survival in japanese renal transplant recipients with Alport syndrome: a retrospective study. BMC Nephrol.

[27] Sugimoto H, Mundel TM, Sund M, Xie L, Cosgrove D, Kalluri R. Bone-marrow-derived stem cells repair basement membrane collagen defects and reverse genetic kidney disease. Proceedings of the National Academy of Sciences. 2006;103(19):7321-6. 10.1073/pnas.0601436103 PMC1464339 16648256

[28] Prodromidi EI, Poulsom R, Jeffery R, Roufosse CA, Pollard PJ, Pusey CD (2006). Bone marrow-derived cells contribute to podocyte regeneration and amelioration of renal disease in a mouse model of Alport syndrome. Stem Cells.

